# Biological characteristics of an enterovirus A71 subgroup C4 strain isolated in China

**DOI:** 10.1186/s12879-025-12241-2

**Published:** 2025-12-04

**Authors:** Tianli Ma, Huan Li, Yunfang Li, Weishi Lin, Zhengying Yu, Lizhong Li, Wei Zhang, Hongbin Song, Leili Jia, Jing Xie

**Affiliations:** 1 Chinese PLA Center for Disease Control and Prevention, 20 Dongda Street, Fengtai District, Beijing, 100071 China; 2 Department of Epidemiology, School of Public Health, China Medical University, Shenyang, China; 3 Southern Medical District of Chinese PLA General Hospital, Beijing, China; 4 The 8th Medical Center of General Hospital of the Chinese People’s Liberation Army, Beijing, China

**Keywords:** Subgroup C4, Viral infectivity and replication, Cytotoxicity, Blood–brain barrier

## Abstract

**Background:**

Hand, foot, and mouth disease (HFMD) is a widespread infectious disease primarily affecting infants and young children. Enterovirus A71 (EV-A71) comprises eight genogroups, among which subgroup C4 is the dominant viral agent in China and is frequently associated with HFMD and central nervous system infections. The genetic characteristics of an EV-A71 subgroup C4 strain obtained in this study were analyzed using whole-genome sequencing. Its biological characteristics, including infectivity, replication, and cytotoxicity, were investigated in human rhabdomyosarcoma (RD) and African green monkey kidney (Vero) cells.

**Methods:**

A clinical EV-A71 C4 subgourp GD10 strain isolated in China was examined to evaluate its genetic and biological features. Its relationships with strains listed in GenBank were evaluated using phylogenetic analysis. Viral infectivity and replication were assessed in RD and Vero cells. Cytotoxicity was evaluated by measuring cell viability, lactate dehydrogenase (LDH) release, and ATP levels. Effects on blood–brain barrier (BBB) integrity were investigated in vitro by assessing transendothelial electrical resistance and viral load across the barrier.

**Results:**

Sequence analysis confirmed that GD10 belonged to subgroup C4 and closely resembled strains from China. GD10 infection induced a pronounced cytopathic effect and elevated viral RNA levels in RD cells but not in Vero cells. The infection time-dependently increased LDH release and reduced ATP levels. GD10 compromised BBB integrity and crossed the cellular barrier in vitro.

**Conclusion:**

The GD10 strain demonstrated strong adaptability to RD cells and impaired BBB function. Our results improve the understanding of virus–host interactions and may support efforts towards EV-A71 vaccine development.

**Clinical Trial:**

Not applicable

**Supplementary Information:**

The online version contains supplementary material available at 10.1186/s12879-025-12241-2.

## Introduction

Enterovirus A71 (EV-A71), a major causative agent of hand, foot, and mouth disease (HFMD), predominantly infects infants and young children. Patients with HFMD often develop rashes on their palms and soles, oral ulcers, and fever [1]. Although HFMD is self-limiting, EV-A71 infection occasionally causes severe neurological complications [2]. EV-A71 was first isolated in 1969 from a child diagnosed with encephalitis in the USA and has since been sporadically reported in America and Europe [3–10]. In 1997, a large HFMD outbreak occurred in Malaysia, causing 34 fatalities [11]. The following year, another extensive outbreak occurred in Taiwan, with over 100,000 reported HFMD cases [12]. Since its emergence, EV-A71 has consistently caused epidemics across the Asia-Pacific region [13–19], causing two outbreaks in Shandong and Anhui provinces between 2007 and 2008 and subsequently becoming endemic in mainland China [20, 21].

EV-A71 currently comprises eight groups based on the nucleotide sequences of *VP1* [22]. Group A includes the prototype strain (BrCr) and several EV-A71 strains that circulated in mainland China in 2008–2010 [3, 23]. Groups B and C are further divided into subgroups B0–B5 and C0–C6, respectively. Subgroups B0–B2 primarily circulated in the Netherlands, Hungary, Bulgaria, and the USA between 1960 and 1989; subgroups B3–B5 circulated in the Asia-Pacific region beginning in 1990 [24–29]. Group C rapidly spread globally, causing numerous outbreaks, particularly across Asia-Pacific countries [11, 12, 30]. Subgroup C4 emerged as the predominant EV-A71 strain in China from 1998 onwards and continues circulating to date [31–33]. Additional genotypes were recently identified; Groups D and G circulated solely in India from 2008, without documented outbreaks [34, 35]. Groups E and F were identified in Africa in 2003 and Madagascar in 2004, respectively [36, 37]. Since HFMD outbreaks were reported in Shandong and Anhui between 2007 and 2008, subgroup C4 of EV-A71 has become the predominant agent circulating in China [20, 21]. Determining the genetic and biological characteristics of the prevalent EV-A71 strain can improve the understanding of its molecular epidemiological characteristics and facilitate the development of effective vaccines. However, few studies have examined the full-genome characteristics of subgroup C4 strains along with their cytopathogenic effects and ability to cross the blood–brain barrier (BBB) in vitro.

In this study, we characterized the genetic properties of an EV-A71 subgroup C4 strain and evaluated its biological characteristics, including infectivity, viral replication, and cytotoxicity in human rhabdomyosarcoma (RD) and African green monkey kidney (Vero) cells. Furthermore, we assessed whether the isolate could infect and cross the BBB using an established in vitro BBB model.

## Materials and methods

### Cell lines and viruses

RD and Vero cells were maintained in Dulbecco’s modified Eagle’s medium (no C11995500BT, Gibco, Grand Island, NY, USA) containing 10% fetal bovine serum (Gibco). The human brain microvessel endothelial cell line (hCMEC/D3) was purchased from Cellverse (iCell-h070; Shanghai, China) and cultured in Dulbecco’s modified Eagle’s medium containing 10% fetal bovine serum and 40 μg/mL endothelial cell growth supplement (E2759; Sigma-Aldrich, St. Louis, MO, USA). The cells were cultured at 37 °C in a 5% CO_2_ incubator. The EV-A71 prototype strain BrCr was preserved in our laboratory. The EV-A71 clinical strain GD10 (GenBank accession number: KJ004559.1) was kindly provided by the Chinese Center for Disease Control and Prevention.

### Viral genome sequencing and phylogenetic analysis

Total viral RNAs were extracted from GD10 strain-infected cell culture supernatants using an RNA extraction kit (RNeasy Mini Kit, 74104; Qiagen, Hilden, Germany), following the manufacturer’s instructions. All mRNAs were subjected to RNA sequencing using an Illumina MiSeq v2 instrument (San Diego, CA, USA) with 300-bp paired-end reads. After removing host-derived reads, virus-specific reads were included in genome alignment and assembly. All sequence reads were mapped to the selected reference EV-A71 virus (list in Table S2) using CLC Bio’s clc_ref_assemble_long program. We obtained the genome of the isolated EV-A71 (GD10 strain). Comparative sequence analyses, including sequence alignments and estimation of genetic distances, were performed using MEGA software (version 11.0; ClustalW, Molecular Evolutionary Genetic Analysis software). Phylogenetic trees were constructed using the neighbor-joining method with Kimura in MEGA, and branch support was calculated based on 1,000 bootstrap replicates.

### Viral infection

Vero and RD cells were seeded into 24-well plates and incubated overnight. Cells at 80% confluence were infected with the BrCr or GD10 strain at a multiplicity of infection (MOI) of 0.01 for 1 h. Following inoculation, the viral suspension was removed, and the cells were washed with phosphate-buffered saline (PBS) before adding fresh culture medium. Cytopathic effect (CPE) was observed, and cell morphology was recorded using a Carl Zeiss Axio Vert.A1 microscope (Oberkochen, Germany). Infected cells were harvested at various time points for RNA extraction for reverse transcription-quantitative PCR (RT-qPCR) and protein extraction for western blotting. The viral titer was measured as the 50% tissue culture infective dose (TCID_50_) per milliliter (mL) according to the Reed and Muench method, as previously described [38].

### RT-qPCR analysis

Infected cells were trypsinized, and total RNA was extracted using an RNA Easy Fast Tissue/Cell Kit (DP451; Tiangen, Shanghai, China). RNA was reverse-transcribed into cDNA using a FastKing RT Kit (KR116; Tiangen, China). RT-qPCR was performed with EV-A71 VP1-specific primers (Table S1) using a SuperReal PreMix Plus (SYBR Green) Kit (FP205; Tiangen, China) on a QuantStudio 3 Real-Time PCR System (Applied Biosystems, Foster City, CA, USA). The RT-qPCR program was performed as follows: predenaturation at 95 °C for 15 min, followed by 40 cycles of denaturation at 95 °C for 10 s, annealing at 60 °C for 20 s and extension at 72 °C for 30 s. EV-A71 expression was normalized to that of GAPDH and presented as the fold-change (EV-A71/GAPDH).

### Cell cytotoxicity and viability assay

Vero and RD cells were seeded into 96-well plates and infected with 0.01 MOI of the BrCr or GD10 strain. Cell proliferation was assessed using a CCK-8 assay kit (40203ES60; Yeasen, Shanghai, China). At the indicated time points post-infection, CCK-8 reagent was added and incubated in the dark. Absorbance was measured at 450 nm using a microplate reader (SYNERGY HTX; BioTek, Winooski, VT, USA). Cell viability was assessed with the CellTiter-Glo Luminescent Cell Viability Assay (G7570; Promega, Madison, WI, USA) based on intracellular ATP quantification. Cell death was evaluated using a CytoTox 96 Non-Radioactive Cytotoxicity Assay (G1780; Promega) by measuring lactate dehydrogenase (LDH) release, following the manufacturer’s instructions.

### Western blotting

Cells were lysed in cold RIPA buffer (P0013B; Beyotime, Shanghai, China) supplemented with protease and phosphatase inhibitors (P1045; Beyotime) on ice, followed by centrifugation at 4 °C. The protein concentration was quantified using a Pierce BCA Protein Assay Kit (23225; Thermo Fisher Scientific, Waltham, MA, USA). Proteins were resolved by 10% polyacrylamide gel electrophoresis and transferred onto polyvinylidene difluoride (PVDF) membranes (0.22 µm, Millipore, Billerica, MA, USA). The membranes were blocked in blocking buffer (PBS containing 5% milk and 0.05% Tween-20) at room temperature, washed with PBST (PBS containing 0.05% Tween-20), and incubated with primary antibodies at a dilution of 1:3000 at 4 °C overnight. After PBST, the membranes were incubated with secondary antibodies at a dilution of 1:5000. The blots were visualized using a SuperSignal West Femto Trial Kit (34094; Thermo Fisher Scientific) and imaged with a ChemiDoc MP Imaging System (Bio-Rad, Hercules, CA, USA). A polyclonal antibody against EV-A71 was generated in-house from rabbits immunized with purified VP1 protein. The VP1 gene was cloned from the BrCr strain by PCR and ligated into the pET24a vector. The recombinant plasmid pET24a-VP1 was transformed into *Escherichia coli* (BL21) cells to induce expression of the target protein. The purified protein was used as immunogen to inoculate rabbits, and the rabbit sera was collected after the fourth immunization. The Rabbit anti-GAPDH (5174S) was purchased from Cell Signaling Technology (Danvers, MA, USA).

### In vitro BBB model and transendothelial electrical resistance (TEER) measurement

hCMEC/D3 cells were seeded on the apical side of Transwell inserts (3462; Corning, Inc., Corning, NY, USA) with hCMEC/D3 complete medium in the upper and lower chambers. Cells were incubated at 37 °C with 5% CO_2_ until confluence, and fresh media was replaced daily. The apical chamber was exposed to the EV-A71 strains at MOI 6.6 for 1 h, then replaced with fresh medium. TEER) was measured using the Millicell^®^-ER system (#MERS00002; Merck Millipore, Kenilworth, NJ, USA), as described previously [39]. Briefly, the electrode was disinfected with 70% ethanol and equilibrated in pre-warmed medium. The longer arm of the electrode was placed in the lower chamber, and the shorter in the upper chamber. Triplicate readings were recorded for each insert. TEER values were calculated by multiplying the average resistance (Ω) by the membrane area (1.12 cm^2^). The background TEER value was used as a mock control.

### Statistical analysis

All experiments were performed with at least three replicates, and data are presented as the means ± standard deviation (SD). GraphPad Prism software (version 8.0.3, GraphPad, Software Inc., San Diego, CA, USA) was used for statistical analysis. A *p-*value < 0.05 was considered statistically significant. The statistical methods are noted in the figure legends.

## Result

### Viral genome and phylogenetic tree

**Fig. 1:**
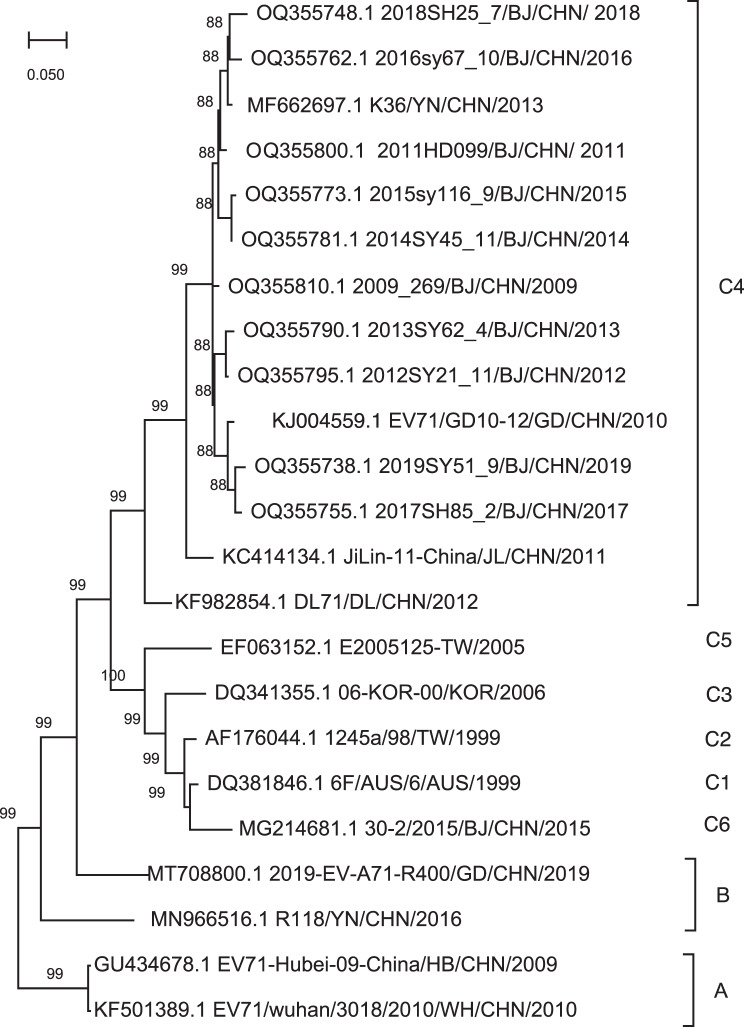
Phylogenetic analysis of enterovirus A71 whole genome sequences. A phylogenetic tree was constructed using the neighbor-joining method with Kimura in MEGA with 1000 bootstrap replicates. The percentage of trees is shown next to the number of branches. The red dots indicate the GD10 strain used in this study

The GD10 strain sequence consisted of 7415 bp and contained a single open reading frame (ORF) encoding a 2193-amino acid polypeptide, deposited in GenBank under accession number KJ004559.1. Phylogenetic analyses were performed to assess genetic relationships between this strain and 22 reference strains isolated worldwide and deposited in GenBank (Table S2). A phylogenetic tree was constructed based on full-genome nucleotide sequences of EV-A71. The GD10 strain shared 88% nucleotide identity with other EV-A71 strains from Beijing, indicating close genetic relatedness and high homology (Fig. 1). These findings suggested that GD10 belong to C4 subgroup and showed high sequence similarity with other previously reported C4 strains in China.

## Infection and replication of the GD10 strain

**Fig. 2:**
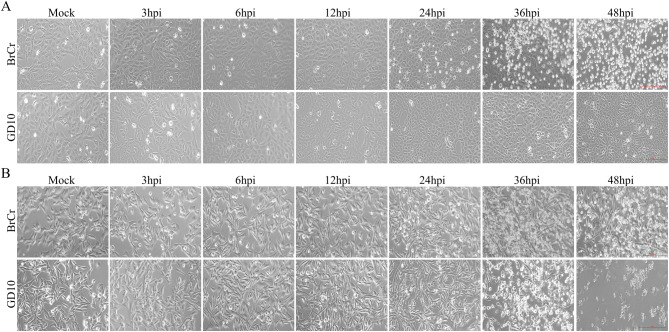
GD10 strain induced CPE in RD cells but not in Vero cells.Vero (**A**) and RD (**B**) cells were infected with the BrCr or GD10 strains at an MOI of 0.01. CPE was examined, and images were captured at different time points after infection. Images are shown at 20× magnification

**Fig. 3:**
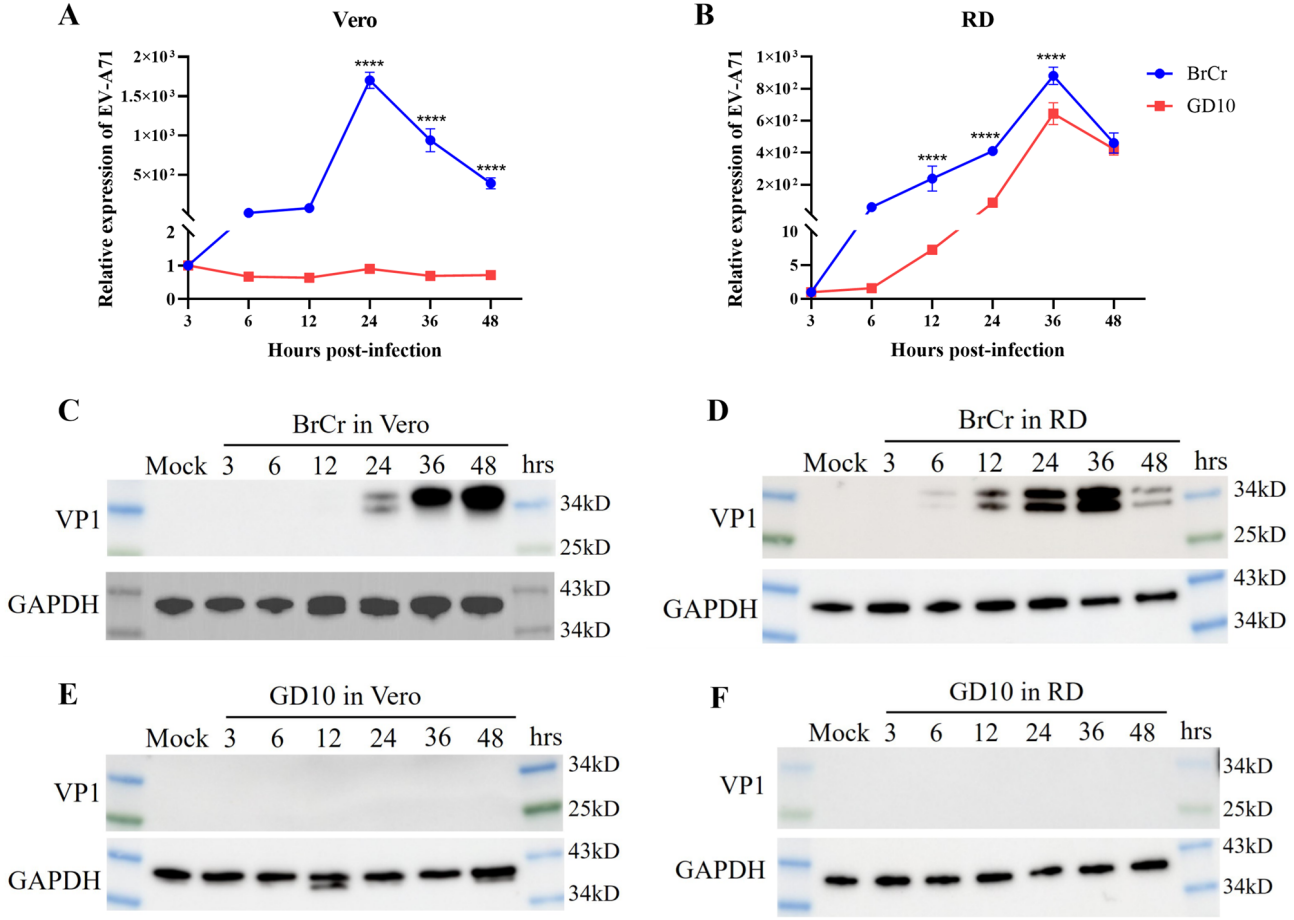
Viral replication and VP1 protein expression of GD10 in Vero and RD cells. Vero and RD cells were infected with BrCr (blue) or GD10 strains (red) at an MOI of 0.01 and harvested at different time points. Viral RNA in the lysates of Vero (**A**) and RD (**B**) cells was examined using RT-qPCR. The expression levels of VP1 protein in the cell lysate were examined by western blotting in Vero (**C**, **E**) and RD (**D**, **F**) cells. All data are shown as the mean values ± sd. Experiments were performed in triplicate and repeated at least twice. Statistical significance was analyzed using two-way ANOVA (*p < 0.05, **p < 0.01, ***p < 0.001, and ****p < 0.0001). Abbreviations: MOI, multiplicity of infection; RD, rhabdomyosarcoma

We initially evaluated CPE in Vero and RD cells to assess the infectivity of the GD10 clinical isolate. Both cell types were infected with the GD10 strain at an MOI of 0.01; the BrCr strain served as a positive control. The BrCr strain induced evident CPE in both cell lines at 36–48 h post-infection (hpi) (Fig. 2). However, GD10 infection induced CPE in RD cells beginning at 36 hpi, whereas no evident cell death occurred in Vero cells. To evaluate replication kinetics, viral RNA expression was measured over time. The BrCr strain replicated robustly in both cell lines (Fig. 3A–B). In RD cells, GD10 replicated significantly but with lower viral RNA levels than those in BrCr (Fig. 3B). However, GD10 RNA was undetectable in Vero cells (Fig. 3A). These results suggest that RD cells more effectively support GD10 replication. We also detected VP1 expression at the protein level using western blotting. For the BrCr strain, VP1 protein was detected as early as 24 hpi in Vero cells but at 6 hpi in RD cells and was found to gradually increase (Fig. 3C–D). However, using antibodies against BrCr VP1 protein, VP1 from the GD10 strain was not detected during infection both within Vero and RD cells (Fig. 3E–F). The inability of the BrCr VP1 antibody to detect GD10 VP1 protein may be related to the antigenic variation of VP1 protein between the BrCr and GD10 strains. Collectively, these data show that GD10 infection induced CPE and support efficient replication in RD cells but not in Vero cells.

## Cell viability and cytotoxicity following GD10 infection

**Fig. 4:**
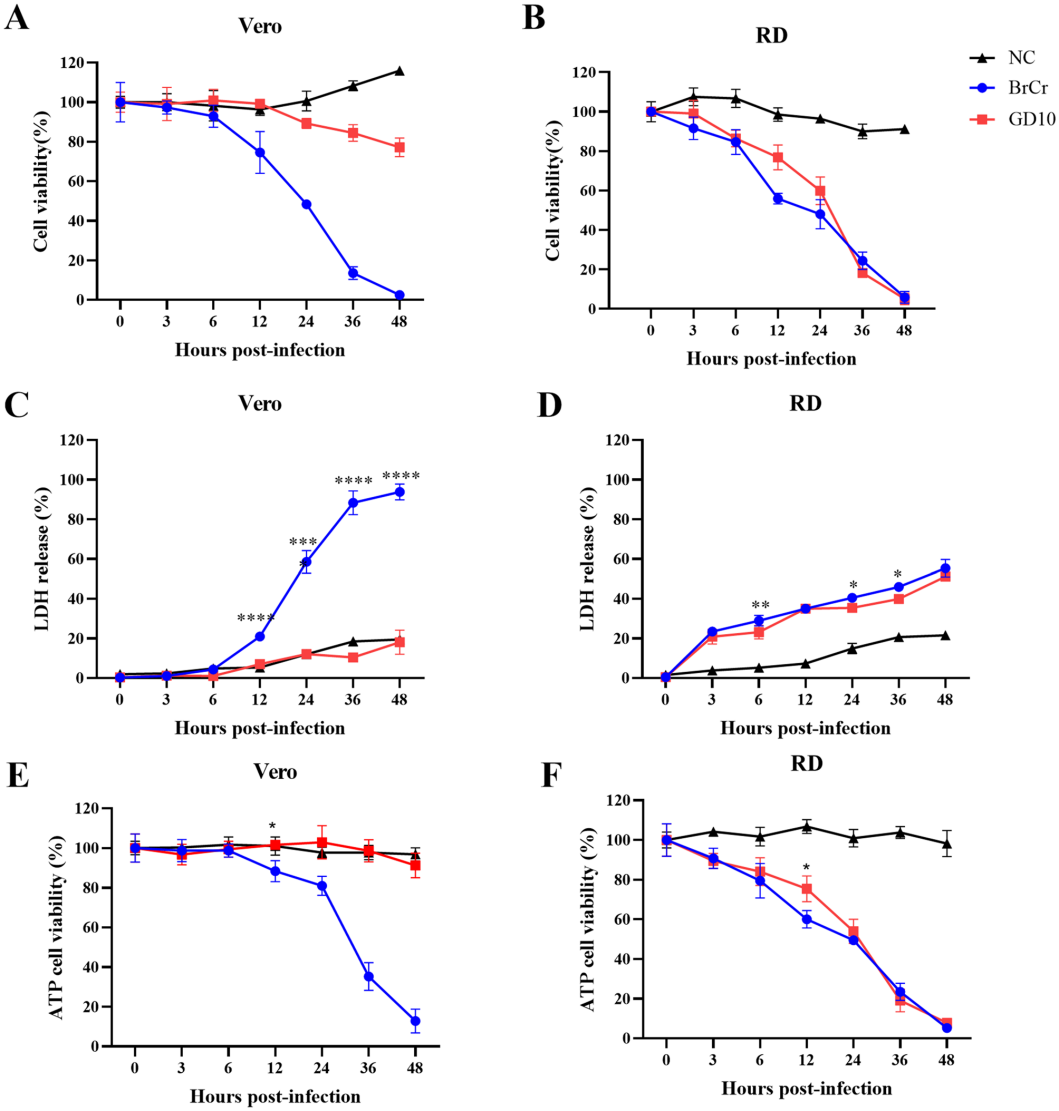
GD10 strain causes cell damage in RD cells. Vero and RD cells were infected with BrCr (blue) or GD10 strains (red) at an MOI of 0.01. Cell viability (**A**, **B**), LDH-release-based cell death (**C**, **D**), and ATP cell viability (**E**, **F**) were assessed at different time points after infection. All data are shown as the mean values ± SD. Experiments were performed in triplicate and repeated at least twice. Statistical significance was analyzed using a two-way ANOVA (*p<0.05,and ***p<0.0001)

Given the observed CPE and viral replication (Figs. 2 and 3), we next assessed the cell death response to GD10 in Vero and RD cells. CCK-8 assay revealed a marked decrease in Vero cell viability following BrCr infection, whereas GD10 did not induce such a decrease (Fig. 4A). In RD cells, both BrCr and GD10 caused significant reductions in viability, consistent with the observed CPE (Fig. 4B). Cytotoxicity was further assessed by measuring LDH release into the supernatant and ATP levels. In Vero cells, BrCr infection led to increased LDH release starting at 12 hpi, with progressive elevation through 48 hpi (Fig. 4C). ATP levels also declined significantly (Fig. 4E). In contrast, GD10 infection did not affect LDH or ATP levels in Vero cells. In RD cells, both BrCr and GD10 increased LDH release and decreased ATP levels (Figs. 4D- 4F). These results indicate that GD10 did not induce detectable cytotoxicity in Vero cells.

## Ability of the GD10 strain to cross the BBB

**Fig. 5:**
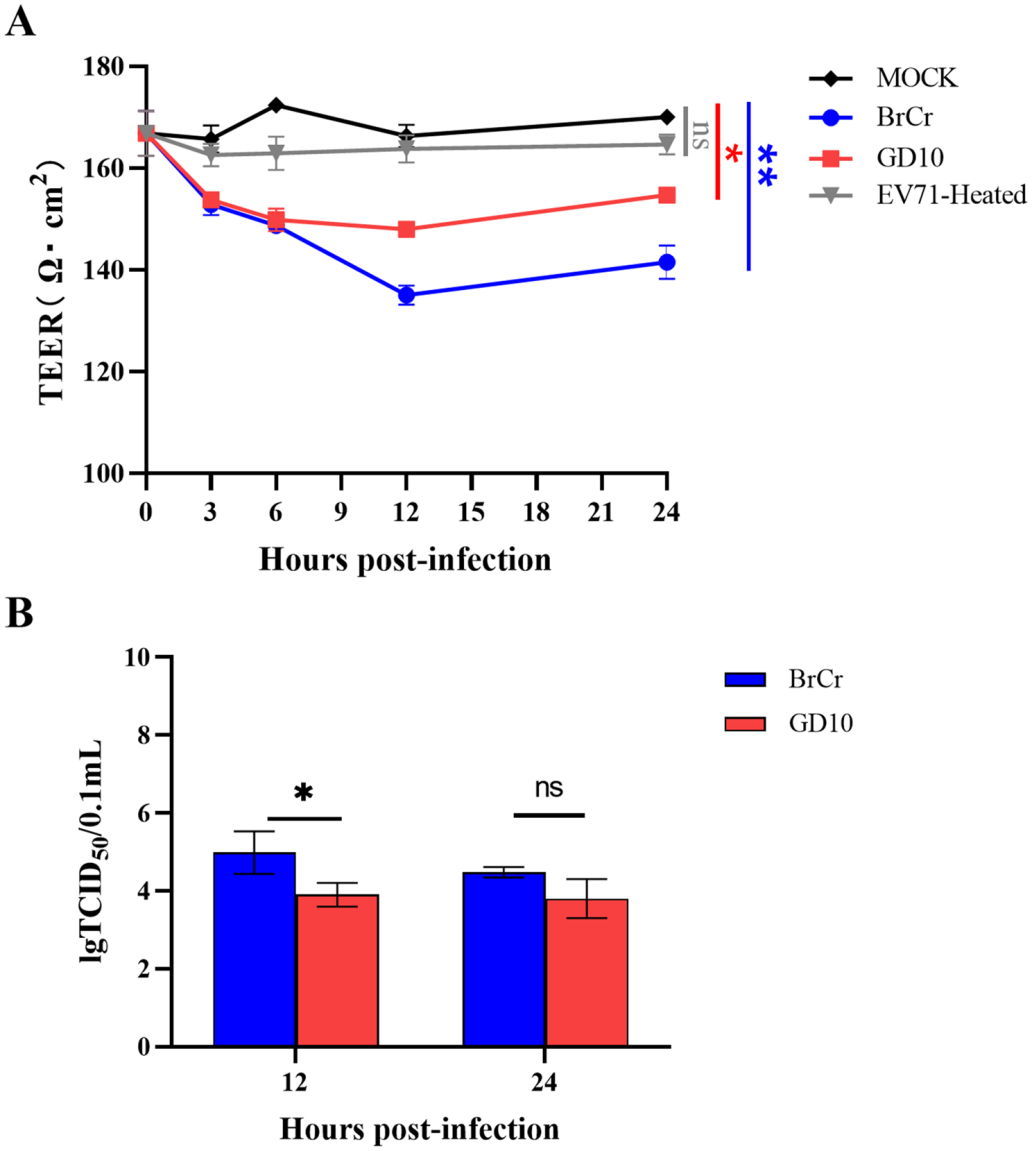
GD10 strain could cross the BBB model in vitro. Human brain microvascular endothelial cells (hCMEC/D3) were cultured in Transwell inserts with growth medium and infected with BrCr (blue), GD10 strain (red), and EV-A71-heated strain (gray) at an MOI of 6.6 for 1 h. Bare PTFE membrane Transwell inserts under the same conditions were used as mock controls. The permeability of the BBB was examined using teer values at different time points after infection (**A**). Statistical analyses were performed using unpaired *t*-tests. The culture medium in the bottom compartment was collected at 12 and 24 hpi, respectively, for quantification of viral load using the TCID_50_ assay (**B**). Statistical significance was determined using one-way ANOVA (*p < 0.05, **p < 0.01). Each assay was performed in triplicate

To assess the effects of GD10 on the integrity of the BBB, we infected hCMEC/D3 cells cultured in Transwell inserts to establish an in vitro BBB model. Both BrCr and GD10 infection significantly reduced TEER relative to that in the mock control from the initial infection stage (Fig. 5A). TEER values progressively declined, reaching a minimum at 12 hpi. Although the BrCr-induced TEER reduction was more pronounced than that induced by GD10, the difference was not significant. Concordantly, inactivated EV-A71 did not affect TEER, indicating that only active infection compromised BBB function. Viral RNA titers using TCID_50_ in the bottom compartment was measured to evaluate BBB penetration (Fig. 5B). At 12 hpi, the BrCr viral titer was significantly higher than that of GD10; however, both strains showed comparable viral titers by 24 hpi (Fig. 5B). These results suggest that GD10 compromised BBB integrity and crossed the barrier early during infection in vitro.

## Discussion

In this study, we examined the genetic diversity and biological characteristics of the clinical EV-A71 subgroup C4 strain GD10. Compared with the BrCr strain, GD10 infection induced severe CPE in RD cells but not in Vero cells. Additionally, RD cells supported GD10 replication, whereas Vero cells did not. EV-A71 reportedly infects various cell lines, including human neuroblastoma (SK-N-SH), colorectal (HT29), neural (SF268), and microvascular endothelial (HMEC-1) cells [40–44]. A previous study showed that five C4 strains from different Chinese regions induce CPE and replicate in Vero cells [45]. However, our findings indicate that Vero cells do not efficiently support GD10 infection. Although BrCr and GD10 exhibited similar replication patterns in RD cells, BrCr viral RNA levels were significantly higher, suggesting a faster replication rate. We did not detect VP1 protein expression in RD cells following GD10 infection, possibly because the anti-EV-A71 antibody could not bind to the GD10 strain owing to the different antigenic epitopes residing in the BrCr strain. We further assessed cell viability and cytotoxicity, demonstrating that GD10 induced cell damage comparable with that caused by BrCr, with increased LDH release and decreased ATP production. These data indicate that GD10 infection induces lytic cell death. Collectively, GD10 exhibits different susceptibility to Vero cells relative to previously reported C4 subgroup strains.

The prototype BrCr strain (genogroup A) was isolated from a patient with central nervous system (CNS) disease in the USA [3]. Subgroup C4 was identified as a major public health concern in China in 2007 [46]. EV-A71-associated HFMD often causes severe neurological complications in children aged < 5 years, indicating that EV-A71 can cross the BBB and invade the CNS [2]. Three subgroup C4 EV-A71 strains induced pathological lesions in neonatal mice and rhesus monkeys [47]. Previously, we showed that GD10 infection caused severe clinical symptoms, including substantial weight loss and hindlimb paralysis, in both BALB/c and C57 mice, confirming its neurovirulence [48]. Using an in vitro model, we demonstrated that GD10 could cross the BBB, as indicated by reduced TEER values. Furthermore, BrCr infection produced greater barrier disruption than GD10, suggesting that BrCr possesses higher neurovirulence.

This study had some limitations. First, we did not compare GD10 with other Chinese subgroup C4 isolates. Second, few cell types were employed to characterize viral biology. Nevertheless, we thoroughly evaluated GD10 infectivity and replication at the cellular level, along with BBB disruption in vitro, facilitating future studies of virus–host interactions and CNS pathogenesis.

In conclusion, we analyzed the genetic and biological properties of a clinically isolated EV-A71 subgroup C4 strain. GD10 exhibited adaptation for infection, replication, and induction of cell death in RD cells and demonstrated BBB penetration in vitro. Our findings improve the understanding of EV-A71 pathogenesis and may inform the development of multivalent EV-A71 vaccines.

## Electronic supplementary material

Below is the link to the electronic supplementary material.

Supplementary Material 1

Supplementary Material 2

Supplementary Material 3

## Data Availability

The data sets generated and/or analyzed during the current study are available from the corresponding author on reasonable request.

## Abbreviations

HFMD: Hand, Foot, and mouth disease
EV-A71: Enterovirus A71
LDH: Lactate dehydrogenase
BBB: Blood–brain barrier
CPE: Cytopathic effect
MOI: Multiplicity of infection
TCID_50_: Tissue culture infective dose 50
TEER: Transendothelial electrical resistance

## References

[1] Tee HK, Zainol MI, Sam I-C, Chan YF. Recent advances in the understanding of enterovirus A71 infection: a focus on neuropathogenesis. Expert Rev Anti Infect Ther. 2021;19:733–47. 10.1080/14787210.2021.1851194 33183118 10.1080/14787210.2021.1851194

[2] Ooi MH, Wong SC, Lewthwaite P, Cardosa MJ, Solomon T. Clinical features, diagnosis, and management of enterovirus 71. Lancet Neurol. 2010;9:1097–105. 10.1016/S1474-4422(10)70209-X 20965438 10.1016/S1474-4422(10)70209-X

[3] Schmidt NJ, Lennette EH, Ho HH. An apparently New enterovirus isolated from patients with disease of the central nervous system. J Infect Dis. 1974;129:304–09. 10.1093/infdis/129.3.304 4361245 10.1093/infdis/129.3.304

[4] Fujimoto T, Yoshida S, Munemura T, Taniguchi K, Shinohara M, Nishio O, et al. Detection and quantification of enterovirus 71 genome from cerebrospinal fluid of an encephalitis patient by PCR applications. Jpn J Infect Dis. 2008;61:497–99. 19050366

[5] Chonmaitree T, Menegus MA, Schervish-Swierkosz EM, Schwalenstocker E. Enterovirus 71 infection: report of an outbreak with two cases of paralysis and a review of the literature. Pediatrics. 1981;67:489–93. 7254970

[6] Alexander JP, Baden L, Pallansch MA, Anderson LJ. Enterovirus 71 infections and neurologic disease–United States, 1977-1991. J Infect Dis. 1994;169:905–08. 10.1093/infdis/169.4.905 8133108 10.1093/infdis/169.4.905

[7] Deibel R, Gross LL, Collins DN. Isolation of a new enterovirus. Exp Biol Med. 1975;148:203–07. 10.3181/00379727-148-38506 10.3181/00379727-148-38506 165523

[8] Blomberg J, Lycke E, Ahlfors K, Johnsson T, Wolontis S, von Zeipel G. Letter: new enterovirus type associated with epidemic of aseptic meningitis and-or hand, foot, and mouth disease. Lancet Lond Engl. 1974;2:112. 10.1016/s0140-6736(74)91684-5 10.1016/s0140-6736(74)91684-5 4136956

[9] Hayward JC, Gillespie SM, Kaplan KM, Packer R, Pallansch M, Plotkin S, et al. Outbreak of poliomyelitis-like paralysis associated with enterovirus 71. Pediatr Infect Dis J. 1989;8:611–15. 10.1097/00006454-198909000-00009 2797956 10.1097/00006454-198909000-00009

[10] Brown BA, Kilpatrick DR, Oberste MS, Pallansch MA. Serotype-specific identification of enterovirus 71 by PCR. J Clin Virol. 2000;16:107–12. 10.1016/s1386-6532(00)00065-2 10720814 10.1016/s1386-6532(00)00065-2

[11] Chan LG, Parashar UD, Lye MS, Ong FG, Zaki SR, Alexander JP, et al. Deaths of children during an outbreak of hand, foot, and mouth disease in sarawak, Malaysia: clinical and pathological characteristics of the disease. For the outbreak study group. Clin Infect Dis Off Publ Infect Dis Soc Am. 2000;31:678–83. 10.1086/314032 10.1086/314032 11017815

[12] Ho M, Chen ER, Hsu KH, Twu SJ, Chen KT, Tsai SF, et al. An epidemic of enterovirus 71 infection in Taiwan. Taiwan Enterovirus Epidemic Work Group. N Engl J Med. 1999;341:929–35. 10.1056/NEJM199909233411301 10.1056/NEJM199909233411301 10498487

[13] Sun L, Zheng H, Zheng H, Guo X, He J, Guan D, et al. An enterovirus 71 epidemic in Guangdong Province of China, 2008: epidemiological, clinical, and virogenic manifestations. Jpn J Infect Dis. 2011;64:13–18. 21266750

[14] Fujimoto T, Chikahira M, Yoshida S, Ebira H, Hasegawa A, Totsuka A, et al. Outbreak of central nervous system disease associated with hand, foot. And Mouth Disease In Jpn Dur The Summer Of 2000: Detect And Mol Epidemiol of Enterovirus 71. Microbiol Immunol. 2002;46:621–27. 10.1111/j.1348-0421.2002.tb02743.x 10.1111/j.1348-0421.2002.tb02743.x 12437029

[15] Jee YM, Cheon D-S, Kim K, Cho JH, Chung Y-S, Lee J, et al. Genetic analysis of the VP1 region of human enterovirus 71 strains isolated in Korea during 2000. Arch Virol. 2003;148:1735–46. 10.1007/s00705-003-0133-6 14505086 10.1007/s00705-003-0133-6

[16] Chan KP, Goh KT, Chong CY, Teo ES, Lau G, Ling AE. Epidemic hand, foot and mouth disease caused by human enterovirus 71, Singapore. Emerg Infect Dis. 2003;9:78–85. 10.3201/eid0901.020112 12533285 10.3201/eid1301.020112 PMC2873753

[17] Ooi MH, Wong SC, Podin Y, Akin W, Sel S del, Mohan A, et al. Human enterovirus 71 disease in Sarawak, Malaysia: a prospective clinical, virological, and molecular epidemiological study. Clin Infect Dis. 2007;44:646–56. 10.1086/511073 17278054 10.1086/511073

[18] Tu P V, Thao NTT, Perera D, Truong KH, Tien NTK, Thuong TC, et al. Epidemiologic and virologic investigation of Hand, foot, and mouth disease, southern Vietnam, 2005. Emerg Infect Dis. 2007;13:1733–41. 10.3201/eid1311.070632 18217559 10.3201/eid1311.070632 PMC3375788

[19] Horwood PF, Andronico A, Tarantola A, Salje H, Duong V, Mey C, et al. Seroepidemiology of human enterovirus 71 infection among children, Cambodia. Emerg Infect Dis. 2016;22:92–95. 10.3201/eid2201.151323 26690000 10.3201/eid2201.151323 PMC4696711

[20] Zhang Y, Tan X-J, Wang H-Y, Yan D-M, Zhu S-L, Wang D-Y, et al. An outbreak of hand, foot, and mouth disease associated with subgenotype C4 of human enterovirus 71 in Shandong, China. J Clin Virol. 2009;44:262–67. 10.1016/j.jcv.2009.02.002 19269888 10.1016/j.jcv.2009.02.002

[21] Zhang Y, Zhu Z, Yang W, Ren J, Tan X, Wang Y, et al. An emerging recombinant human enterovirus 71 responsible for the 2008 outbreak of hand foot and mouth disease in Fuyang city of China. Virol J. 2010;7. 10.1186/1743-422x-7-94 10.1186/1743-422X-7-94 PMC2885340 20459851

[22] Kinobe R, Wiyatno A, Artika IM, Safari D. Insight into the enterovirus A71: a review. Rev Med Virol. 2022;32. 10.1002/rmv.2361 10.1002/rmv.2361 35510476

[23] Yu H, Chen W, Chang H, Tang R, Zhao J, Gan L, et al. Genetic analysis of the VP1 region of enterovirus 71 reveals the emergence of genotype a in central China in 2008. Virus Genes. 2010;41:1–4. 10.1007/s11262-010-0472-9 10.1007/s11262-010-0472-9 20306124

[24] Nagy G, Takátsy S, Kukán E, Mihály I, Dömök I. Virological diagnosis of enterovirus type 71 infections: experiences gained during an epidemic of acute CNS diseases in Hungary in 1978. Arch Virol. 1982;71:217–27. 10.1007/bf01314873 6285858 10.1007/BF01314873

[25] Chumakov M, Voroshilova M, Shindarov L, Lavrova I, Gracheva L, Koroleva G, et al. Enterovirus 71 isolated from cases of epidemic poliomyelitis-like disease in Bulgaria. Arch Virol. 1979;60:329–40. 10.1007/bf01317504 228639 10.1007/BF01317504

[26] Van Der Sanden S, Koopmans M, Uslu G, Van Der Avoort H. Epidemiology of enterovirus 71 in the Netherlands, 1963 to 2008. J Clin Microbiol. 2009;47:2826–33. 10.1128/jcm.00507-09 19625480 10.1128/JCM.00507-09 PMC2738086

[27] Van Der Sanden S, Van Der Avoort H, Lemey P, Uslu G, Koopmans M. Evolutionary trajectory of the VP1 gene of human enterovirus 71 genogroup B and C viruses. J Gen Virol. 2010;91:1949–58. 10.1099/vir.0.019695-0 20375223 10.1099/vir.0.019695-0

[28] Liu Y, Zhang F, Fu C, Wu S, Chen X, Shi Y, et al. Combination of intratypic and intertypic recombinant events in EV71: a novel evidence for the “triple-recombinant” strains of genotype a viruses in Mainland China from 2008 to 2010. Virus Genes. 2015;50:365–74. 10.1007/s11262-015-1170-4 25724176 10.1007/s11262-015-1170-4

[29] Liu Y, Zhou J, Ji G, Gao Y, Zhang C, Zhang T, et al. A novel subgenotype C6 enterovirus A71 originating from the recombination between subgenotypes C4 and C2 strains in mainland China. Sci Rep. 2022;12:593. 10.1038/s41598-021-04604-x 35022489 10.1038/s41598-021-04604-x PMC8755819

[30] Komatsu H, Shimizu Y, Takeuchi Y, Ishiko H, Takada H. Outbreak of severe neurologic involvement associated with enterovirus 71 infection. Pediatr Neurol. 1999;20:17–23. 10.1016/s0887-8994(98)00087-3 10029254 10.1016/s0887-8994(98)00087-3

[31] Zhang Y, Wang J, Guo W, Wang H, Zhu S, Wang D, et al. Emergence and transmission pathways of rapidly evolving evolutionary branch C4a strains of human enterovirus 71 in the Central Plain of China. PLoS One. 2011;6:e27895. 10.1371/journal.pone.0027895 22125635 10.1371/journal.pone.0027895 PMC3220707

[32] Zhang Y, Tan X, Cui A, Mao N, Xu S, Zhu Z, et al. Complete genome analysis of the C4 subgenotype strains of enterovirus 71: predominant recombination C4 viruses persistently circulating in China for 14 years. PLoS One. 2013;8:e56341. 10.1371/journal.pone.0056341 23441179 10.1371/journal.pone.0056341 PMC3575343

[33] Guan D, Van Der Sanden S, Zeng H, Li W, Zheng H, Ma C, et al. Population Dynamics and genetic diversity of C4 strains of human enterovirus 71 in Mainland China, 1998–2010. PLoS One. 2012;7:e44386. 10.1371/journal.pone.0044386 22984501 10.1371/journal.pone.0044386 PMC3440427

[34] Rao CD, Yergolkar P, Shankarappa KS. Antigenic diversity of Enteroviruses associated with nonpolio acute flaccid paralysis, India, 2007–2009. Emerg Infect Dis. 2012;18:1833–40. 10.3201/eid1811.111457 23092622 10.3201/eid1811.111457 PMC3559176

[35] Mohanty MC, Varose SY, Rane SV, Pawar SD, Tandale BV. Seroprevalence of enterovirus 71 among children in Western India. Viruses. 2025;17:356. 10.3390/v17030356 40143284 10.3390/v17030356 PMC11946791

[36] Fernandez-Garcia MD, Kebe O, Fall AD, Dia H, Diop OM, Delpeyroux F, et al. Enterovirus A71 Genogroups C and E in children with acute flaccid paralysis, West Africa. Emerg Infect Dis. 2016;22:753–55. 10.3201/eid2204.151588 26982072 10.3201/eid2204.151588 PMC4806963

[37] Bessaud M, Razafindratsimandresy R, Nougairède A, Joffret M-L, Deshpande JM, Dubot-Pérès A, et al. Molecular comparison and evolutionary analyses of VP1 nucleotide sequences of new African human enterovirus 71 isolates reveal a wide genetic diversity. PLoS One. 2014;9:e90624. 10.1371/journal.pone.0090624 24598878 10.1371/journal.pone.0090624 PMC3944068

[38] Reed L, Muench H. A simple method of estimating fifty percent endpoints. Am J Hyg. 1938;27:493–97.

[39] Stone NL, England TJ, O’Sullivan SE. A novel transwell blood brain barrier Model using primary human cells. Front Cell Neurosci. 2019;13. 10.3389/fncel.2019.00230 10.3389/fncel.2019.00230 PMC6563620 31244605

[40] Fu F, Zhao J, Xi X. Identification of genes involved in enterovirus 71 infected SK-N-SH cells. Int J Clin Exp Pathol. 2017;10:11588–95. 31966515 PMC6966083

[41] Shih S-R, Stollar V, Lin J-Y, Chang S-C, Chen G-W, Li M-L. Identification of genes involved in the host response to enterovirus 71 infection. J Neurovirol. 2004;10:293–304. 10.1080/13550280490499551 15385252 10.1080/13550280490499551

[42] Lui YLE, Timms P, Hafner LM, Tan TL, Tan KH, Tan EL. Characterisation of enterovirus 71 replication kinetics in human colorectal cell line, HT29. SpringerPlus. 2013, 2. 10.1186/2193-1801-2-267 23875129 10.1186/2193-1801-2-267 PMC3696168

[43] Lu J. Viral kinetics of enterovirus 71 in human abdomyosarcoma cells. World J Gastroenterol. 2011;17:4135. 10.3748/wjg.v17.i36.4135 22039330 10.3748/wjg.v17.i36.4135 PMC3203367

[44] Liang C, Sun M, Lei H, Chen S, Yu C, Liu C, et al. Human endothelial cell activation and apoptosis induced by enterovirus 71 infection. J Med Virol. 2004;74:597–603. 10.1002/jmv.20216 10.1002/jmv.20216 15484266

[45] Wang L, Tang S, Li Y, Zhao H, Dong C, Cui P, et al. A comparison of the biological characteristics of EV71 C4 subtypes from different epidemic strains. Virol Sin. 2010;25:98–106. 10.1007/s12250-010-3102-8 10.1007/s12250-010-3102-8 PMC8227828 20960306

[46] Nayak G, Bhuyan SK, Bhuyan R, Sahu A, Kar D, Kuanar A. Global emergence of enterovirus 71: a systematic review. Beni-Suef Univ J Basic Appl Sci. 2022;11. 10.1186/s43088-022-00258-4 10.1186/s43088-022-00258-4 PMC9188855 35730010

[47] Zhongping X, Hua L, Ting Y, Zhengling L, Min F, Tianhong X, et al. Biological characteristics of different epidemic enterovirus 71 strains and their pathogeneses in neonatal mice and rhesus monkeys. Virus Res. 2016;213:82–89. 10.1016/j.virusres.2015.11.007 26555165 10.1016/j.virusres.2015.11.007

[48] Xie J, Hu X, Li H, Zhu H, Lin W, Li L, et al. Murine models of neonatal susceptibility to a clinical strain of enterovirus A71. Virus Res. 2023;324:199038. 10.1016/j.virusres.2022.199038 36599394 10.1016/j.virusres.2022.199038 PMC10194309

