# Supplementary material for: Biological characteristics of an enterovirus A71 subgroup C4 strain isolated in China
Source: BMC Infect Dis. 2025 Dec 4;26:19. doi: 10.1186/s12879-025-12241-2 (PMC12781644; doi:10.1186/s12879-025-12241-2)
Supplement: Supplementary file 1 — Supplementary Material 1 [file 12879_2025_12241_MOESM1_ESM.pdf]

Fig.3C

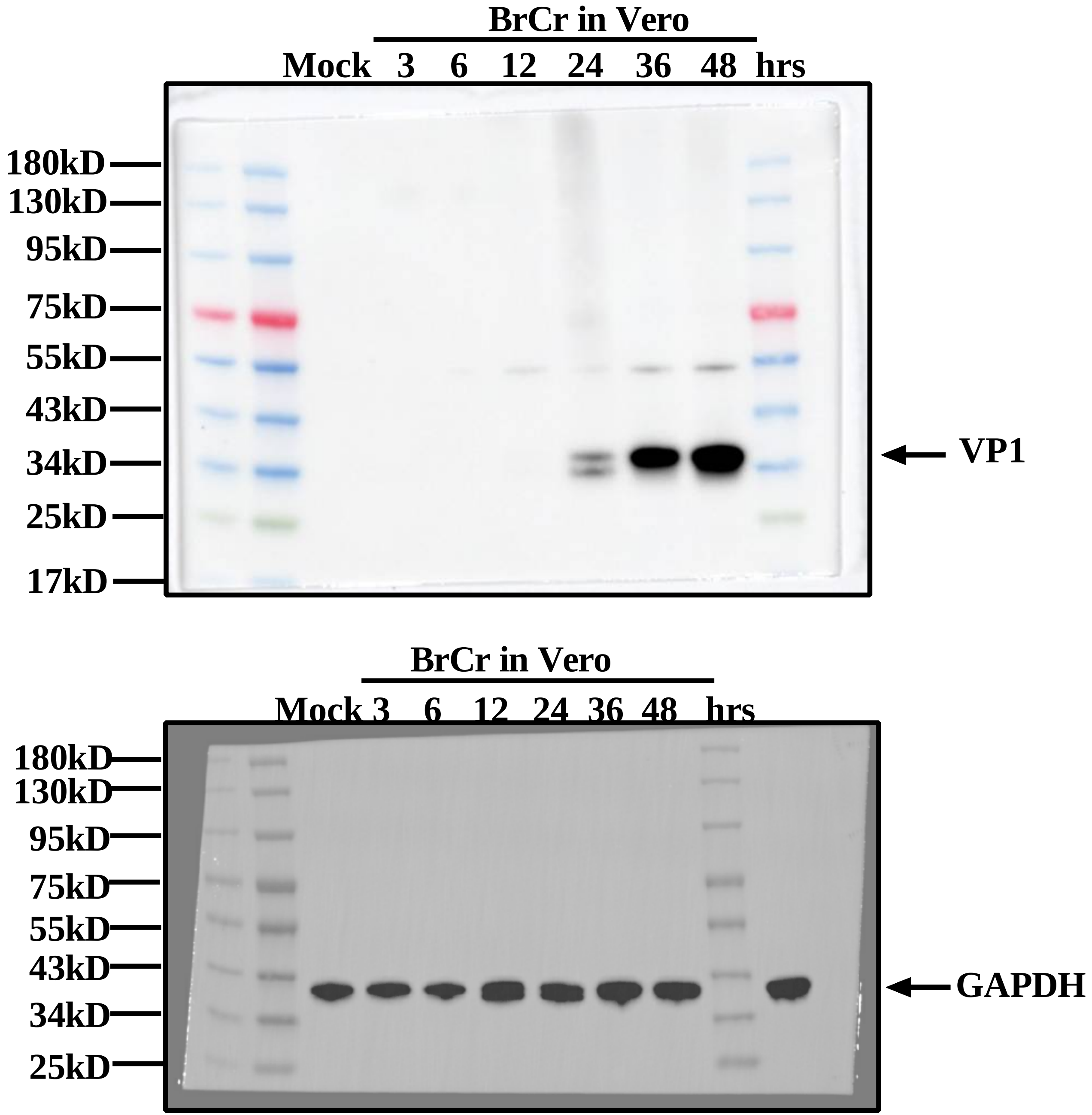

BrCr in Vero
Mock
3
6
12
24
36
48
hrs
180kD
130kD
95kD
75kD
55kD
43kD
34kD
25kD
17kD
VP1
BrCr in Vero
Mock
3
6
12
24
36
48
hrs
180kD
130kD
95kD
75kD
55kD
43kD
34kD
25kD
GAPDH

Fig.3D

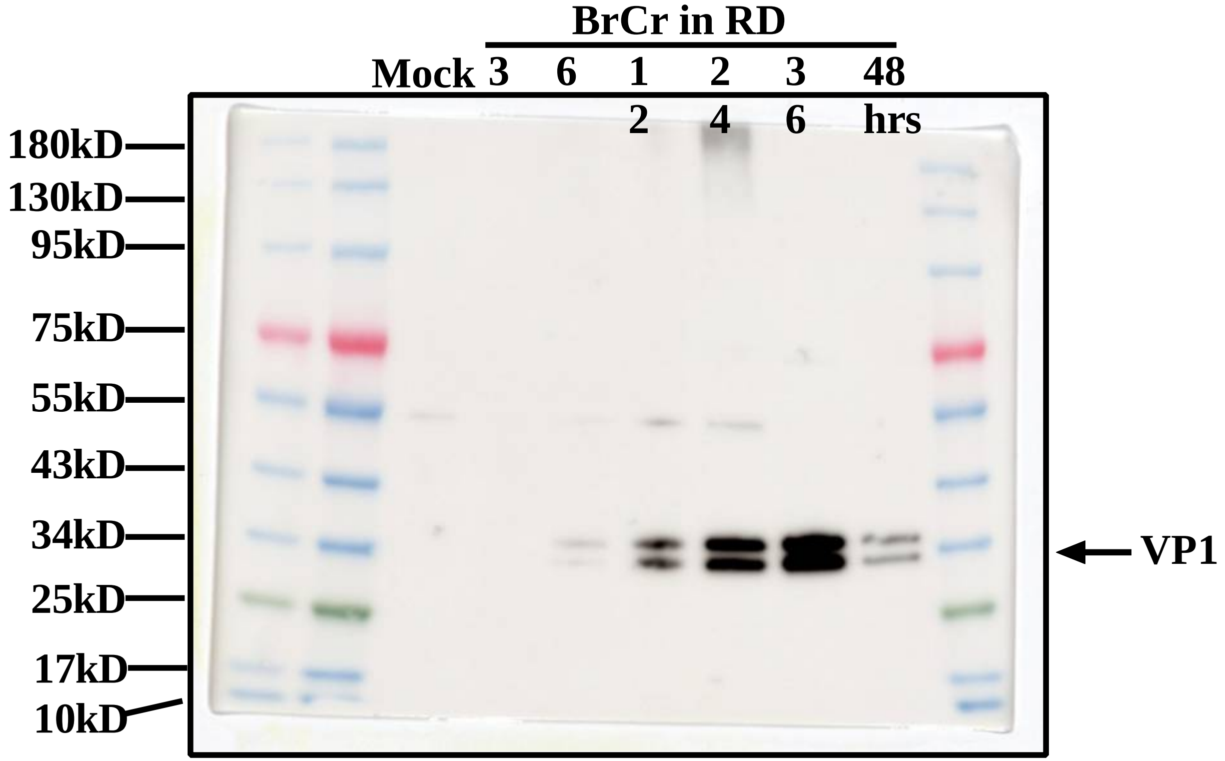
BrCr in RD
Mock 3 6 12 24 36 48 hrs
180kD
130kD
95kD
75kD
55kD
43kD
34kD
25kD
17kD
10kD
VP1

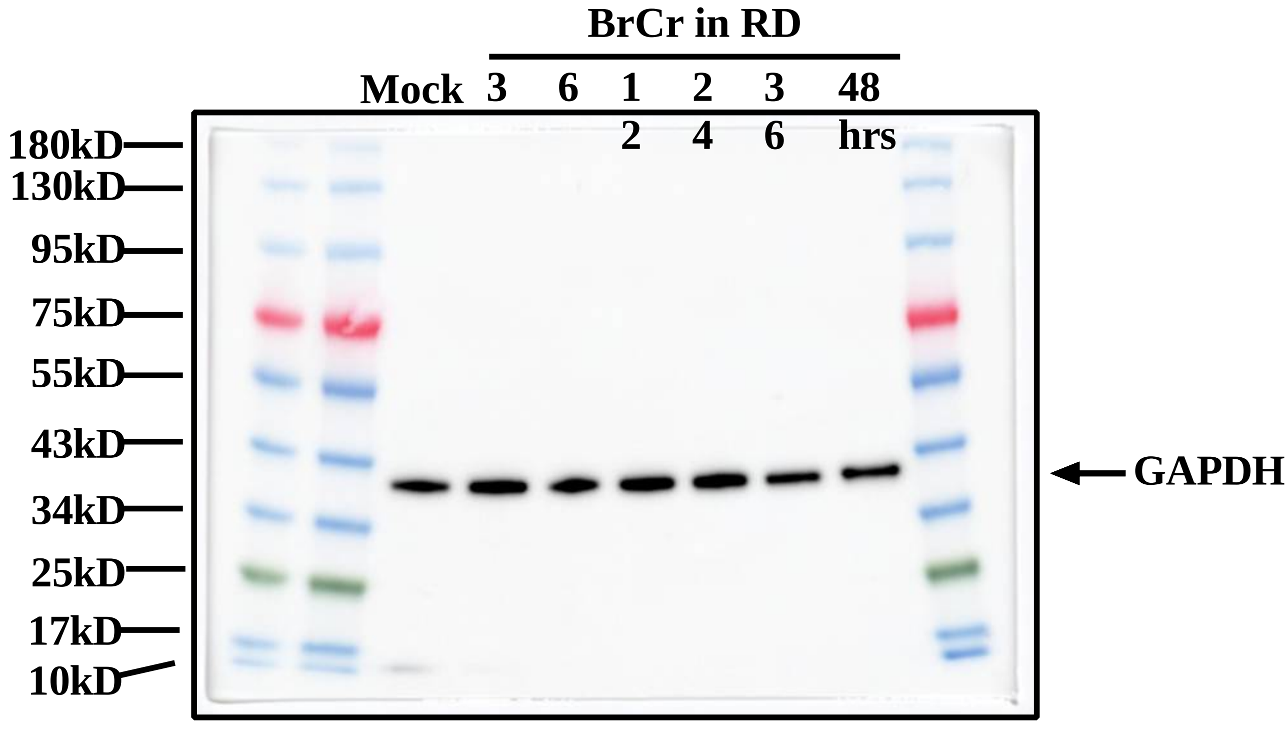
BrCr in RD
Mock 3 6 12 24 36 48 hrs
180kD
130kD
95kD
75kD
55kD
43kD
34kD
25kD
17kD
10kD
GAPDH

Fig.3E

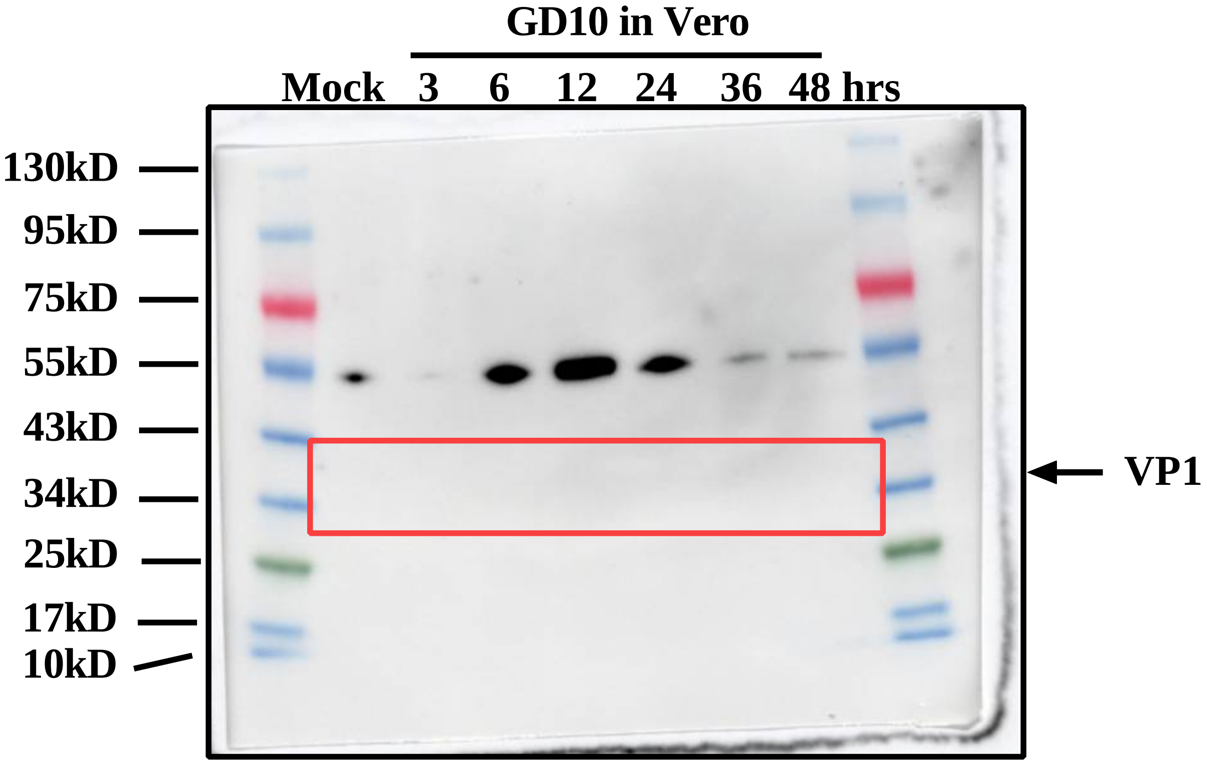
GD10 in Vero
Mock 3 6 12 24 36 48 hrs
130kD
95kD
75kD
55kD
43kD
34kD
25kD
17kD
10kD
VP1

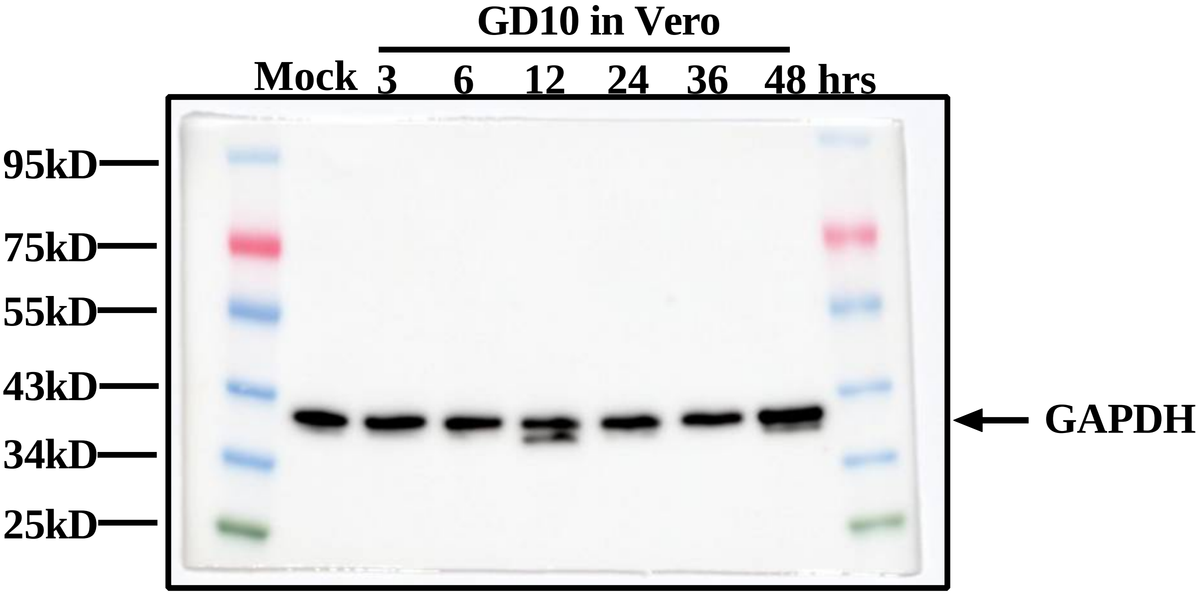
GD10 in Vero
Mock 3 6 12 24 36 48 hrs
95kD
75kD
55kD
43kD
34kD
25kD
GAPDH

Fig.3F

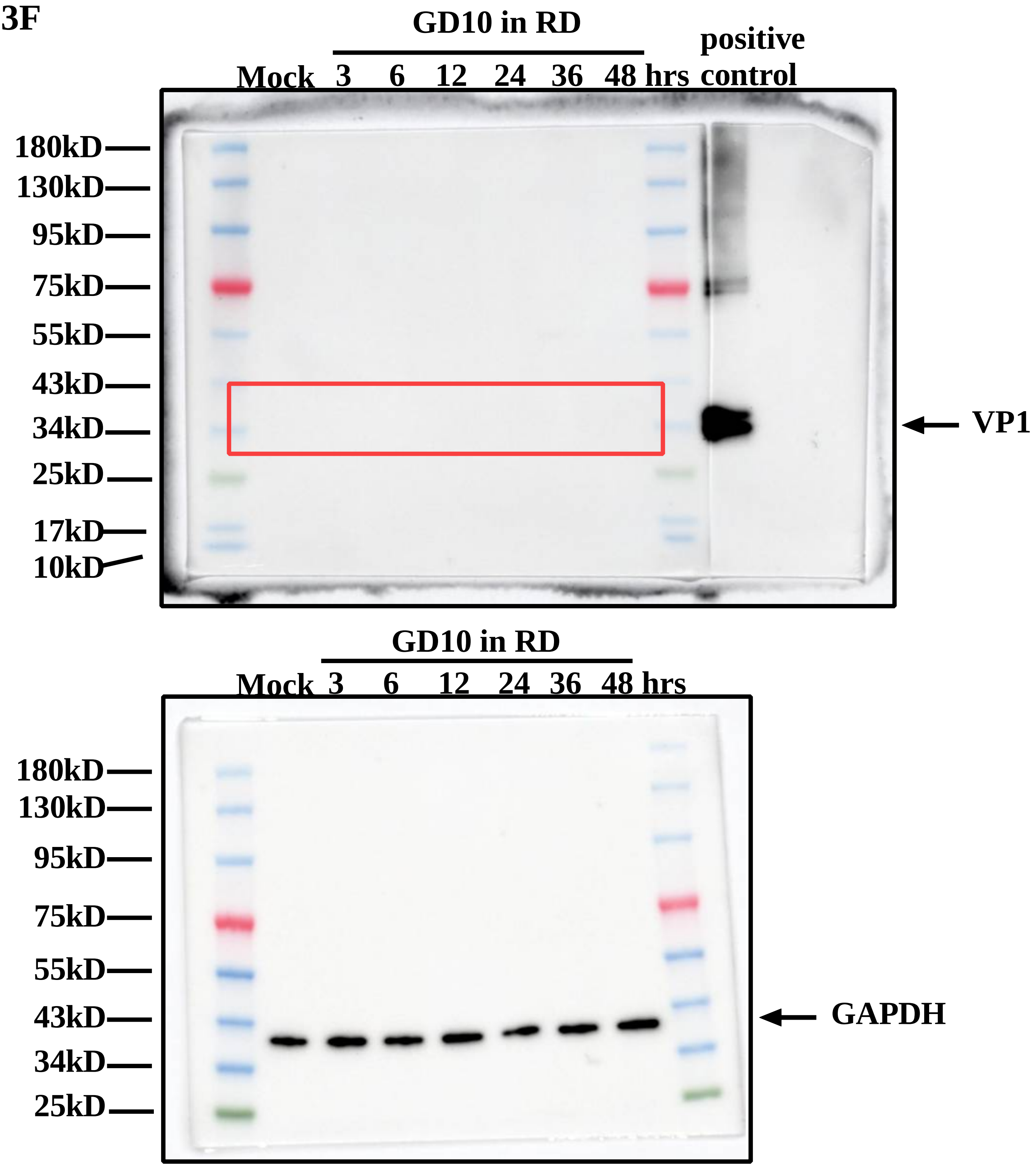

GD10 in RD
Mock
3
6
12
24
36
48 hrs
positive control
180kD
130kD
95kD
75kD
55kD
43kD
34kD
25kD
17kD
10kD
VP1
GD10 in RD
Mock
3
6
12
24
36
48 hrs
180kD
130kD
95kD
75kD
55kD
43kD
34kD
25kD
GAPDH
