# Supplementary material for: Biological characteristics of an enterovirus A71 subgroup C4 strain isolated in China
Source: BMC Infect Dis. 2025 Dec 4;26:19. doi: 10.1186/s12879-025-12241-2 (PMC12781644; doi:10.1186/s12879-025-12241-2)
Supplement: Supplementary file 2 — Supplementary Material 2 [file 12879_2025_12241_MOESM2_ESM.docx]

**Table S1.** Primer sequences for real-time qPCR assay.

| **Primer** | **Sequence（5'-3'）** |
| --- | --- |
| EV71-F | CCAATCTCAGCGGCTTGGAG |
| EV71-R | CACTCAAGCTCTACCGGCAC |
| GAPDH-F | ACCACAGTCCATGCCATCAC |
| GAPDH-R | TCCACCACCCTGTTGCTGTA |
