# Supplementary material for: Biological characteristics of an enterovirus A71 subgroup C4 strain isolated in China
Source: BMC Infect Dis. 2025 Dec 4;26:19. doi: 10.1186/s12879-025-12241-2 (PMC12781644; doi:10.1186/s12879-025-12241-2)
Supplement: Supplementary file 3 — Supplementary Material 3 [file 12879_2025_12241_MOESM3_ESM.docx]

**Table S2.** Strain name, accession numbers, genogroups, the year and the place of isolation information of the EV-A71 strains used to generate the phylogenetic analysis in this study.

| **Strain name** | **Accession numbers** | **Genogroups** | **The year of isolation** | **The place of isolation** |
| --- | --- | --- | --- | --- |
| EV71-Hubei-09-China | GU434678.1 | A | 2009 | CHN/Hubei |
| [EV71/wuhan/3018/2010](https://www.ncbi.nlm.nih.gov/nuccore/KF501389.1/) | KF501389.1 | A | 2010 | CHN/Wuhan |
| [R118/YN/CHN/2016](https://www.ncbi.nlm.nih.gov/nuccore/MN966516.1/) | MN966516.1 | B | 2016 | CHN/Yunnan |
| [2019-EV-A71-R400](https://www.ncbi.nlm.nih.gov/nuccore/MT708800.1/) | MT708800.1 | B | 2019 | CHN/Guangdong |
| 6F/AUS/6/99 | DQ381846.1 | C1 | 1999 | Australia |
| 1245a/98/tw | AF176044.1 | C2 | 1999 | Taiwan |
| 06-KOR-00 | DQ341355.1 | C3 | 2006 | South Korea |
| DL71 | KF982854.1 | C4 | 2012 | CHN/Dalian |
| 2018SH25_7/BJ/China | OQ355748.1 | C4 | 2018 | CHN/Beijing |
| 2016sy67_10/BJ/China | OQ355762.1 | C4 | 2016 | CHN/Beijing |
| K36/YN/CHN/2013 | MF662697.1 | C4 | 2013 | CHN/Yunnan |
| 2011HD099/BJ/China | OQ355800.1 | C4 | 2011 | CHN/Beijing |
| 2015sy116_9/BJ/China | OQ355773.1 | C4 | 2015 | CHN/Beijing |
| 2014SY45_11/BJ/China | OQ355781.1 | C4 | 2014 | CHN/Beijing |
| 2009_269/BJ/China | OQ355810.1 | C4 | 2009 | CHN/Beijing |
| 2013SY62_4/BJ/China | OQ355790.1 | C4 | 2013 | CHN/Beijing |
| 2012SY21_11/BJ/China | OQ355795.1 | C4 | 2012 | CHN/Beijing |
| EV71/GD10-12/2010 | KJ004559.1 | C4 | 2012 | CHN/Beijing |
| 2019SY51_9/BJ/China | OQ355738.1 | C4 | 2019 | CHN/Beijing |
| 2017SH85_2/BJ/China | OQ355755.1 | C4 | 2017 | CHN/Beijing |
| JiLin-11-China | KC414134.1 | C4 | 2011 | CHN/Jilin |
| E2005125-TW | EF063152.1 | C5 | 2005 | Taiwan |
| 30-2/2015/BJ | MG214681.1 | C6 | 2015 | CHN/Beijing |
